# Supplementary material for: Contrary neuronal recalibration in different multisensory cortical areas
Source: eLife. 2023 Mar 6;12:e82895. doi: 10.7554/eLife.82895 (PMC9988259; doi:10.7554/eLife.82895)
Supplement: Figure 2—source data 1. [file elife-82895-fig2-data1.doc]

**Figure 2–source data 1: Individual monkey summary statistics of behavioral shifts**

|  | | | **Monkey K** | **Monkey B** | **Monkey D** | **Pooled** |
| --- | --- | --- | --- | --- | --- | --- |
| **Δ^+^** | **N** | | 44 | 36 | 161 | 241 |
|  | **Vestibular** | **PSE shift** | 1.27° ± 0.21° | 1.06° ± 0.52° | 1.09° ± 0.13° | 1.12° ± 0.12° |
|  |  | **p** | 5.0 × 10^-7^ *** | 0.048 * | 2.2 × 10^-14^ *** | 2.3 × 10^-17^ *** |
|  | **Visual** | **PSE shift** | -1.11° ± 0.22° | 0.36° ± 0.49° | -0.87° ± 0.09° | -0.73° ± 0.11° |
|  |  | **p** | 9.0 × 10^-6^ *** | 0.47 | 2.1 × 10^-17^ *** | 6.5 × 10^-11^ *** |
| **Δ^-^** | **N** | | 41 | 35 | 151 | 227 |
|  | **Vestibular** | **PSE shift** | -0.37° ± 0.28° | -1.55° ± 0.44° | -2.18° ± 0.15° | -1.76° ± 0.14° |
|  |  | **p** | 0.19 | 1.2 × 10^-3^ ** | 5.9 × 10^-31^ *** | 1.0 × 10^-28^ *** |
|  | **Visual** | **PSE shift** | 0.88° ± 0.25° | 2.25° ± 0.37° | 0.89° ± 0.09° | 1.10° ± 0.10° |
|  |  | **p** | 1.2 × 10^-3^ ** | 7.7 × 10^-7^ *** | 3.3 × 10^-18^ *** | 5.4 × 10^-23^ *** |

N = number of sessions. PSE shifts = mean ± SEM. P-values from paired t-test. *** p < 0.001; ** p < 0.01; * p < 0.05
