# Supplementary material for: Contrary neuronal recalibration in different multisensory cortical areas
Source: eLife. 2023 Mar 6;12:e82895. doi: 10.7554/eLife.82895 (PMC9988259; doi:10.7554/eLife.82895)
Supplement: Figure 3—source data 1. [file elife-82895-fig3-data1.doc]

**Figure 3–source data 1: Individual monkey summary statistics for MSTd correlations**

|  | | **Monkey D** | **Monkey K** | **Pooled** |
| --- | --- | --- | --- | --- |
| **Vestibular** | **r** | 0.97 | 0.54 | 0.62 |
|  | **p** | 0.17 | 0.086 | 0.019 * |
|  | **N** | 3 | 11 | 14 |
| **Visual** | **r** | 0.66 | 0.35 | 0.38 |
|  | **p** | 0.02 * | 0.017 * | 2.7 × 10^-3^ *** |
|  | **N** | 12 | 47 | 59 |

N = number of neurons, r and p-values from Pearson correlations. *** p < 0.001; * p < 0.05.
