# Supplementary material for: Contrary neuronal recalibration in different multisensory cortical areas
Source: eLife. 2023 Mar 6;12:e82895. doi: 10.7554/eLife.82895 (PMC9988259; doi:10.7554/eLife.82895)
Supplement: Figure 3—source data 2. [file elife-82895-fig3-data2.docx]

**Figure 3–source data 2: Comparison of pooled model (PM) and linear mixed model (LMM) for MSTd**

|  | | **Model values** | | | | |
| --- | --- | --- | --- | --- | --- | --- |
|  |  | β | p | SE | AIC | BIC |
| **Vestibular** | **PM** | 6.14 | 0.019 * | 2.26 | 122 | 124 |
|  | **LMM** | 7.03 | 0.029 * | 2.64 | 128 (+6) | 132 (+8) |
| **Visual** | **PM** | 2.13 | 0.003 ** | 0.68 | 437 | 443 |
|  | **LMM** | 2.93 | 0.033 * | 1.33 | 442 (+5) | 455 (+12) |

Model values: β (the regression coefficient between neuronal and perceptual shifts), associated p-value and standard error (SE) of β. AIC: Akaike Information Criterion. BIC: Bayesian information criterion. Lower (AIC and BIC) values indicate a preferred model, within a given condition (vestibular or visual). Values in parenthesis indicate the difference (LMM − PM). ** p < 0.01; * p < 0.05.
