# Supplementary material for: Contrary neuronal recalibration in different multisensory cortical areas
Source: eLife. 2023 Mar 6;12:e82895. doi: 10.7554/eLife.82895 (PMC9988259; doi:10.7554/eLife.82895)
Supplement: Figure 4—source data 1. [file elife-82895-fig4-data1.doc]

**Figure 4–source data 1: Individual monkey summary statistics for PIVC correlations**

|  | | **Monkey D** | **Monkey B** | **Pooled** |
| --- | --- | --- | --- | --- |
| **Vestibular** | **r** | 0.81 | 0.86 | 0.80 |
|  | **p** | 3.8 × 10^-6^ *** | 5.6 × 10^-3^ *** | 9.7 × 10^-8^ *** |
|  | **N** | 22 | 8 | 30 |
| **Visual** | **r** | 0.47 | - | 0.26 |
|  | **p** | 0.24 | - | 0.47 |
|  | **N** | 8 | 2 | 10 |

N = number of neurons, r and p-values from Pearson correlations. *** p < 0.001.
