# Supplementary material for: Contrary neuronal recalibration in different multisensory cortical areas
Source: eLife. 2023 Mar 6;12:e82895. doi: 10.7554/eLife.82895 (PMC9988259; doi:10.7554/eLife.82895)
Supplement: Figure 4—source data 2. [file elife-82895-fig4-data2.docx]

**Figure 4–source data 2: Comparison of pooled model (PM) and linear mixed model (LMM) for PIVC**

|  | | **Model values** | | | | |
| --- | --- | --- | --- | --- | --- | --- |
|  |  | β | p | SE | AIC | BIC |
| **Vestibular** | **PM** | 5.38 | 9.7 × 10^-8^ *** | 0.76 | 223 | 227 |
|  | **LMM** | 5.78 | 1.7 × 10^-6^ *** | 1.23 | 227 (+4) | 236 (+9) |
| **Visual** | **PM** | 2.11 | 0.47 | 2.78 | 74 | 75 |
|  | **LMM** | -2.58 | 0.75 | 7.59 | 80 (+6) | 82 (+7) |

Model values: β (the regression coefficient between neuronal and perceptual shifts), associated p-value and standard error (SE) of β. AIC: Akaike Information Criterion. BIC: Bayesian information criterion. Lower (AIC and BIC) values indicate a preferred model, within a given condition (vestibular or visual). Values in parenthesis indicate the difference (LMM − PM). *** p < 0.001.
