# Supplementary material for: Contrary neuronal recalibration in different multisensory cortical areas
Source: eLife. 2023 Mar 6;12:e82895. doi: 10.7554/eLife.82895 (PMC9988259; doi:10.7554/eLife.82895)
Supplement: Figure 5—source data 1. [file elife-82895-fig5-data1.doc]

**Figure 5–source data 1: Individual monkey summary statistics for VIP correlations**

|  | | **Monkey D** | **Monkey B** | **Pooled** |
| --- | --- | --- | --- | --- |
| **Vestibular** | **r** | 0.76 | 0.81 | 0.77 |
|  | **p** | 3.2 × 10^-6^ *** | 8.8 × 10^-3^ *** | 2.7× 10^-8^ *** |
|  | **N** | 28 | 9 | 37 |
| **Visual** | **r** | -0.69 | -0.73 | -0.68 |
|  | **p** | 2.2 × 10^-6^ *** | 0.16 | 8.4× 10^-7^ *** |
|  | **N** | 37 | 5 | 42 |

N = number of neurons, r and p-values from Pearson correlations. '***': p < 0.001.
