# Supplementary material for: Contrary neuronal recalibration in different multisensory cortical areas
Source: eLife. 2023 Mar 6;12:e82895. doi: 10.7554/eLife.82895 (PMC9988259; doi:10.7554/eLife.82895)
Supplement: Figure 5—source data 2. [file elife-82895-fig5-data2.docx]

**Figure 5–source data 2: Comparison of pooled model (PM) and linear mixed model (LMM) for VIP**

|  | | **Model values** | | | | |
| --- | --- | --- | --- | --- | --- | --- |
|  |  | β | p | SE | AIC | BIC |
| **Vestibular** | **PM** | 4.62 | 2.7× 10^-8^ *** | 0.65 | 291 | 296 |
|  | **LMM** | 4.61 | 1.5× 10^-8^ *** | 0.68 | 297 (+6) | 307 (+11) |
| **Visual** | **PM** | -7.75 | 8.4× 10^-7^ *** | 1.33 | 323 | 329 |
|  | **LMM** | -7.55 | 5.3× 10^-7^ *** | 1.33 | 329 (+6) | 340 (+11) |

Model values: β (the regression coefficient between neuronal and perceptual shifts), associated p-value and standard error (SE) of β. AIC: Akaike Information Criterion. BIC: Bayesian information criterion. Lower (AIC and BIC) values indicate a preferred model, within a given condition (vestibular or visual). Values in parenthesis indicate the difference (LMM − PM). *** p < 0.001.
